# The associations of herpes simplex virus and varicella zoster virus infection with dementia: a nationwide retrospective cohort study

**DOI:** 10.1186/s13195-024-01418-7

**Published:** 2024-03-12

**Authors:** Eunhae Shin, Sang Ah Chi, Tae-Young Chung, Hee Jin Kim, Kyunga Kim, Dong Hui Lim

**Affiliations:** 1 Department of Ophthalmology, Samsung Medical Center, Sungkyunkwan University School of Medicine, Seoul, South Korea; 2 Department of Health Sciences and Technology, Samsung Advanced Institute for Health Sciences & Technology, Sungkyunkwan University, Seoul, South Korea; 3 Biomedical Statistics Center, Research Institute for Future Medicine, Samsung Medical Center, Seoul, South Korea; 4 Department of Neurology, Samsung Medical Center, Sungkyunkwan University School of Medicine, Seoul, South Korea; 5 Neuroscience Center, Research Institute for Future Medicine, Samsung Medical Center, Samsung Alzheimer Research Center, Samsung Medical Center, Seoul, South Korea; 6 Department of Digital Health, Samsung Advanced Institute for Health Sciences & Technology, Sungkyunkwan University, Seoul, South Korea; 7 Samsung Advanced Institute for Health Science & Technology, Sungkyunkwan University School of Medicine, Seoul, South Korea

**Keywords:** Herpes simplex virus (HSV), Varicella zoster virus (VZV), Dementia, Subtype

## Abstract

**Background:**

In this study, the risk of dementia in patients with a history of herpes simplex virus (HSV) or varicella zoster virus (VZV) infection was evaluated.

**Methods:**

This nationwide cohort study used data from the Korean National Health Insurance Service collected between 2006 and 2017. A total of 752,205 subjects ≥ 45 years of age not diagnosed with dementia until 2006 were included. A multivariate Cox regression model, adjusted for age, sex, and other comorbidities, was used to assess the hazard ratio (HR) for dementia based on VZV or HSV infection. The interaction effects of both viral infections were analysed. Viral infections are classified into four categories: eye, central nervous system (CNS), simple, and complicated. The hazard ratio (HR) of viral infection was analysed based on the type of dementia.

**Results:**

In multivariable analysis, both HSV and VZV infection were associated with an increased risk of dementia (HR = 1.38, 95% confidence interval, CI:1.33–1.43) and (HR = 1.41, 95% CI:1.37–1.46), respectively. Patients who experienced both HSV and VZV infections were also at an increased risk of dementia (HR = 1.57, 95% CI:1.50–1.63). The co-infection group showed the shortest time from viral infection to dementia diagnosis (4.09 ± 3.02 years). In the subgroup analysis, all types of HSV and VZV infections were associated with an increased risk of dementia compared to the non-infection group. The eye, CNS, and complicated VZV infections were associated with a significantly higher risk than simple VZV infections. There were no significant differences between the subtypes of HSV infection. Furthermore, HSV, VSV, and co-infection were associated with an increased risk of all dementia types, including Alzheimer’s disease (AD) and vascular dementia (VD).

**Conclusions:**

Individual HSV and VZV infections were associated with an increased risk of all types of dementia, including AD and VD. Patients co-infected with HSV and VZV, VZV infection in the eye, CNS, or complicated type were more vulnerable to the development of dementia.

**Supplementary Information:**

The online version contains supplementary material available at 10.1186/s13195-024-01418-7.

## Background

Dementia is the clinical diagnosis of acquired progressive decline in multiple cognitive domains requiring new functional dependence [1, 2]. With an ageing global population, dementia is becoming a major social burden. According to the Global Burden of Disease Study 2015, Alzheimer’s disease (AD) and other dementias accounted for 38.2% of deaths globally, causing over 1.9 million deaths in 2015 [3]. Therefore, exploring the risk factors for dementia has significant clinical value.

Several microbial pathogens, including viruses, bacteria, fungi, and protozoa, can increase inflammation and susceptibility to AD [4]. After penetration to human body, the herpes simplex virus (HSV) resides dorsal root ganglia and the autonomic nervous system. When activated, HSV could invades the brain and destroy it [5]. This inflammation to neuro-system is considered as key component of AD [6, 7]. The association between the HSV and dementia has been previously investigated. Seropositive HSV infection has been shown to cause cognitive impairment and increase the risk of developing AD [8, 9]. Additionally, HSV infection in individuals with the type-4 allele of apolipoprotein E significantly increases the risk of sporadic AD [10]. The varicella zoster virus (VZV) is a member of the *Herpesviridae* family. In a laboratory study of primary human spinal astrocytes, VZV infection was shown to induce amylin expression and potentially lead to cognitive impairment [11]. Another laboratory finding suggested that subsequent infection of VZV after HSV-1 would provoke the reactivation of HSV-1 and lead to accumulation of amyloid-β and P-tau that are found in AD [12].

Population-based cohort studies have suggested that HSV or VZV infection is associated with an increased risk of dementia. A registry-based cohort study in Sweden found that HSV infection without treatment increased the risk of dementia [adjusted hazard ratio (HR) = 1.50)] [13]. Another study conducted in Taiwan based on the National Health Insurance Research Database claimed that VZV was associated with an increased risk of dementia (HR = 1.11) [14]. Additionally, a cohort study conducted in South Korea revealed that patients with VZV had a higher risk of dementia (adjusted HR = 1.12) [15].

However, other studies reported contradictory results. A study on serum HSV1 immunoglobulin level concluded that HSV1 seropositivity was not associated with risk of dementia [7]. A multicentre observational cohort study showed heterogeneous results among four cohorts regarding the association between untreated herpes virus infection and dementia [16]. A cohort study in the United Kingdom found no association between VZV and dementia [17]. Similarly, a comparative cohort study using national Danish data did not show an increased risk of dementia in patients with VZV [18].

As found in previous studies, it is inconclusive whether these viral infections have a definite association with an increased risk of dementia. A recent meta-analysis described that HSV-1 infection was associated with increased risk of AD [19]. However, it pointed out that among included studies, high-quality group showed higher odds ratio than that of the moderate-quality group. In this sense, well-organized study would show clear association between viral infection and dementia.

In this nationwide study with a large cohort, the association between HSV and VZV infection and dementia was comprehensively investigated. The risks of single-infection and co-infection based on the infection site were analysed. Furthermore, the risk was assessed based on the type of dementia associated with each viral infection.

## Methods

### Data source and study setting

The Korean National Health Insurance Service (NHIS) is the only health insurer in Korea and provides a mandatory universal medical insurance system to 97% of the Korean population, with the Medical Aid programme financed by the government for the 3% of the population in the lowest income bracket. The NHIS collects clinical records of the diagnoses and prescriptions for each patient. The NHIS also provides national health screening for all Koreans aged > 40 years and all employees, regardless of age. The results of general health examinations and questionnaires on lifestyle behaviours are also recorded.

The NHIS databank contains databases compiling patient data on qualifications (e.g., age, sex, income, region, and type of eligibility), claims [general information on specification, consultation statements, diagnosis statements defined by the International Classification of Disease 10th revision (ICD-10), and prescription statements], health check-ups self-questionnaire on health behaviour (e.g., past medical history, smoking, and drinking), anthropometric measurements [e.g., body mass index (BMI) and blood pressure], and laboratory test results (e.g., fasting glucose and lipid levels), and mortality [20, 21].

This nationwide population-based retrospective cohort study used authorised data from the Korean NHIS database. The study was conducted in accordance with the tenets of the Declaration of Helsinki and was approved by the Institutional Review Board of the Samsung Medical Center (IRB File No. SMC 2018-08-017). The review board waived the requirement for written informed consent owing to the publicly available and anonymous nature of the data and the retrospective nature of the study.

### Study population

**Fig. 1:**
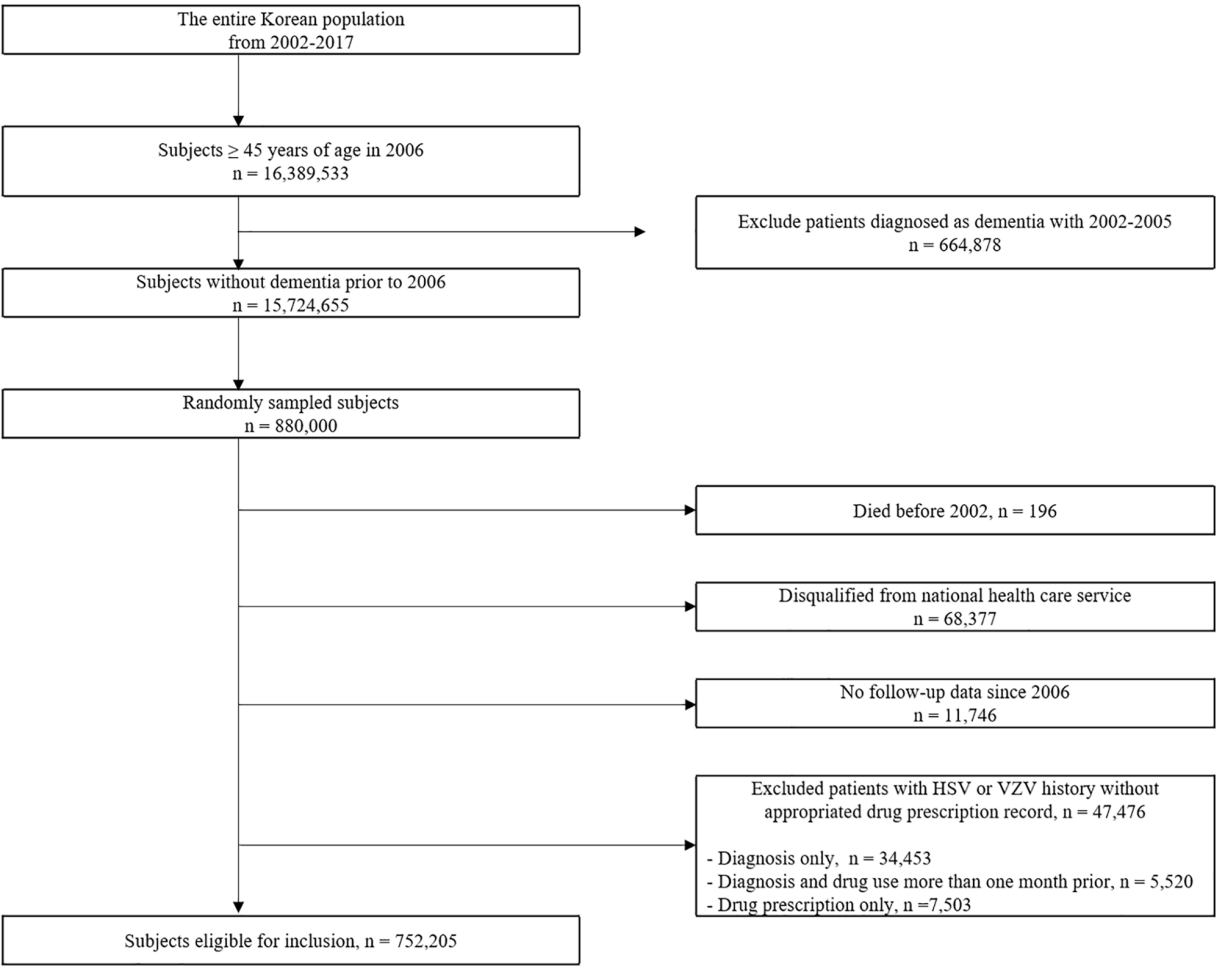
Flow chart of study population

Data of the Korean population with clinical and health screening records from 1 January 2002 to 31 December 2017 were collected from the NHIS database. Data from patients who were > 45 years old in 2006 and who had not been diagnosed with dementia between 2002 and 2005 were collected. Of 15,724,655 individuals, 880,000 were randomly sampled. Individuals who died before 2002 (*n* = 196) and those who were disqualified from the NHIS between 2002 and 2005 were excluded (*n* = 68,377). Additionally, individuals with no follow-up records since 2006 were excluded (*n* = 11,746). Patients diagnosed with HSV or VZV without taking antiviral medication within one month were excluded (*n* = 47,476). Finally, a total of 752,205 individuals were included in the analysis (Fig. 1).

### Exposure variables

Each viral infection was defined as any of the ICD-10 diagnostic codes for HSV and VZV infection combined with the prescription of any systemic or topical antiviral drug within one month of diagnosis. Each diagnosis was subdivided into four categories: eye, central nervous system (CNS), complicated, and simple skin and genital infection. Detailed ICD-10 diagnostic codes are provided in Supplementary Tables 1, and working definitions are shown in Supplementary Table 2. If the patient had several viral episodes with different codes, a diagnosis with specific localisation, including the eye and CNS, was considered.

### Study outcomes and follow-up

The outcome variable was newly developed dementia, defined as the ICD-10 diagnostic code for dementia (F00, F01, F02, F03, G30, or G31.00), and at least one of the dementia medicine prescriptions [acetylcholinesterase inhibitors (donepezil hydrochloride, rivastigmine, and galantamine) or N-methyl-D-aspartate receptor antagonist (memantine)]. The ICD-10 codes for dementia are presented in Supplementary Table (1) To investigate the effects of viral infections according to the subtypes of dementia, AD and vascular dementia (VD) was defined. AD occurrence was defined as the first prescription of an acetylcholinesterase inhibitor or NMDA receptor antagonist with an ICD-10 code for AD (F00 or G30), while vascular dementia (VD) was defined as the first prescription of the same medication with an ICD-10 code for VD (F01). An additional ICD-10 code for unspecified dementia (F03) was permitted for each AD or VD category. The definitions of each type of dementia are presented in Supplementary Table (2) The study follow-up period was from 1 January 2006 to 31 December 2017 or until the diagnosis of dementia.

### Other covariables

Possible confounding factors were comprehensively assessed: age, sex, BMI, household income level, and comorbidities [hypertension (HTN), diabetes mellitus (DM), dyslipidaemia, chronic kidney disease (CKD), ischaemic stroke, coronary heart disease (CHD), and depression] diagnosed in 2006 using ICD-10 codes. This methodology was adapted from previously published nationwide epidemiologic studies [22–26]. The ICD-10 codes used to define the variables and working definitions are presented in Supplementary Tables 1 and 2. Age was classified into two categories: (1) under 65 years and (2) 65 years and over. BMI was defined as weight in kilograms divided by the square of height in metres (kg/m^2^) and was divided into four categories: (1) underweight: < 18.5 kg/m^2^; (2) normal:18.5 to < 23 kg/m^2^ (normal); (3) overweight:23 to < 25 kg/m^2^; and (4) obese: ≤ 25 kg/m^2^. Household income levels were derived from national health insurance premiums. The insurance premium was divided into 20 levels, and participants were categorised into four groups based on the level: (1) low: levels 1–5; (2) lower-middle: levels 6–10; (3) higher-middle: levels 11–15; and (4) high: levels 16–20.

### Statistical analyses

Continuous variables are presented as mean ± standard deviation (SD), and categorical variables are presented as numbers and percentages. The Student’s *t*-test and chi-square test were used to calculate the statistical differences between continuous and categorical variables, respectively. Univariate Cox proportional hazards regression analysis was used to calculate the crude HR and 95% confidence intervals (CI) for dementia. Multivariate Cox proportional hazards regression analysis was performed after adjusting for potential confounders. HSV and VZV were considered time-varying variables to reflect changes in status during the follow-up period. Based on the subdivisions of exposure, pairwise HR of dementia according to different references were calculated. Statistical significance was set at *p* < 0.05. All statistical analyses were performed using SAS (version 9.3; SAS Institute Inc., Cary, NC, USA) and R Statistical Software (version 3.5; Foundation for Statistical Computing, Vienna, Austria).

## Results

### Baseline characteristics of the study population

The participants’ characteristics are presented in Table 1. All the factors listed in Table 1 showed statistically significant differences between the dementia and non-dementia groups (*p* < 0.05). Participants in the dementia group were older than those in the non-dementia group (age, mean ± SD:73.0 ± 8.5 years vs. 57.8 ± 10.2 years). The percentage of females was higher in the dementia group (69.5% vs. 52.5%). The proportions of participants with depression, dyslipidaemia, ischaemic stroke, CHD, HTN, DM, and CKD were higher in the dementia group (Table 1).

**Table 1:** Baseline characteristics of cohort population by presence of dementia

Variables		Cohort (*N* = 752,205)	No dementia (*N* = 699,826)	Dementia (*N* = 52,379)	*p*-value
Follow-up period (years)		10.85 ± 2.79	11.12 ± 2.57	7.26 ± 3.10	-
Age (years)		58.8 ± 10.8	57.8 ± 10.2	73.0 ± 8.5	< 0.0001
	< 65	537,743 (71.5%)	530,107 (75.7%)	7,636 (14.6%)	< 0.0001
	≥ 65	214,462 (28.5%)	169,719 (24.3%)	44,743 (85.4%)	
Sex	Male	348,125 (46.3%)	332,169 (47.5%)	15,956 (30.5%)	< 0.0001
	Female	404,080 (53.7%)	367,657 (52.5%)	36,423 (69.5%)	
HSV infection	None	589,529 (78.4%)	544,963 (77.9%)	44,566 (85.1%)	< 0.0001
	Total	162,676 (21.6%)	154,863 (22.1%)	7,813 (14.9%)	
	Eye	17,659 (2.3%)	16,627 (2.4%)	1,032 (2%)	
	CNS	64 (0%)	60 (0%)	4 (0%)	
	Complicated	11,310 (1.5%)	10,903 (1.6%)	407 (0.8%)	
	Simple	133,643 (17.8%)	127,273 (18.2%)	6,370 (12.2%)	
VZV infection	None	567,874 (75.5%)	526,176 (75.2%)	41,698 (79.6%)	< 0.0001
	Total	184,331 (24.5%)	173,650 (24.8%)	10,681 (20.4%)	
	Eye	4,687 (0.6%)	4,388 (0.6%)	299 (0.6%)	
	CNS	45,515 (6.1%)	42,568 (6.1%)	2,947 (5.6%)	
	Complicated	35,503 (4.7%)	33,421 (4.8%)	2,082 (4%)	
	Simple	98,626 (13.1%)	93,273 (13.3%)	5,353 (10.2%)	
HSV or VZV infection	No	475,884 (63.3%)	438,832 (62.7%)	37,052 (70.7%)	< 0.0001
	HSV only	91,990 (12.2%)	87,344 (12.5%)	4,646 (8.9%)	
	VZV only	113,645 (15.1%)	106,131 (15.2%)	7,514 (14.3%)	
	Co-infection	70,686 (9.4%)	67,519 (9.6%)	3,167 (6%)	
Depression		58,961 (7.8%)	51,704 (7.4%)	7,257 (13.9%)	< 0.0001
Dyslipidaemia		137,800 (18.3%)	124,673 (17.8%)	13,127 (25.1%)	< 0.0001
Ischaemic stroke		30,950 (4.1%)	25,086 (3.6%)	5,864 (11.2%)	< 0.0001
Coronary heart disease		86,082 (11.4%)	75,653 (10.8%)	10,429 (19.9%)	< 0.0001
Hypertension		239,976 (31.9%)	210,783 (30.1%)	29,193 (55.7%)	< 0.0001
Diabetes mellitus		157,642 (21%)	139,387 (19.9%)	18,255 (34.9%)	< 0.0001
Chronic kidney disease		3,287 (0.4%)	2,913 (0.4%)	374 (0.7%)	< 0.0001
Body mass index	Underweight	15,993 (2.1%)	13,916 (2%)	2,077 (4%)	< 0.0001
	Normal	220,444 (29.3%)	206,375 (29.5%)	14,069 (26.9%)	
	Overweight	169,660 (22.6%)	160,748 (23%)	8,912 (17%)	
	Obese	226,268 (30.1%)	214,846 (30.7%)	11,422 (21.8%)	
	not applicable	119,840 (15.9%)	103,941 (14.9%)	15,899 (30.4%)	
Household income level	Low	138,623 (18.4%)	129,529 (18.5%)	9,094 (17.4%)	< 0.0001
	Lower middle	136,824 (18.2%)	128,962 (18.4%)	7,862 (15%)	
	Higher middle	181,648 (24.1%)	170,718 (24.4%)	10,930 (20.9%)	
	High	255,307 (33.9%)	237,910 (34%)	17,397 (33.2%)	
	not applicable	39,803 (5.3%)	32,707 (4.7%)	7,096 (13.5%)	

*Student’s t-test for continuous variables, chi-square test for discrete variables

VZV, varicella zoster virus; HSV, herpes simplex virus; CNS, central nervous system

The mean follow-up period of the entire cohort was 10.85 ± 2.79 years (median, 12.01 years). The mean age of each group is: 58.8 ± 10.8 years in total cohort, 59.2 ± 11.4 years in participants without viral infection, 56.9 ± 9.2 years in HSV infection, 59.5 ± 10.1 years in VZV infection, and 57.9 ± 9.1 years in co-infection. The age of dementia diagnosis did not vary according to the viral infection: 5.02 ± 3.22 years after HSV infection vs. 4.93 ± 3.20 years after VZV infection. However, the period was shorter in cases of co-infection (4.09 ± 3.02 years). The mean follow-up period to dementia diagnosis was 7.06 ± 3.15 years in participants without viral infection, 8.63 ± 2.75 years in the HSV group, 8.50 ± 2.76 years in the VZV group, and 9.25 ± 2.49 years in the co-infection group.

### Association of viral infection and dementia

In univariate analysis (Supplementary Table 3), the HSV-only, VZV-only, and co-infection groups were associated with an increased risk of dementia (HR = 1.16, 95% CI:1.13–1.20), (HR = 1.62, 95% CI:1.58–1.66), and (HR = 1.54, 95% CI:1.49–1.60) respectively. In the multivariate analysis (Table 2), the HSV-only, VZV-only, and co-infection groups were all associated with an increased risk of dementia (HR = 1.38, 95% CI:1.33–1.43), (HR = 1.41, 95% CI:1.37–1.46), and (HR = 1.57, 95% CI:1.50–1.63), respectively.

**Table 2:** Impact of viral infection on the risk of dementia via multivariate time-varying cox regression analyses

Variables		Cohort (*N* = 752,205)	All dementia		Alzheimer’s disease		Vascular dementia	
			Hazard ratio (95% CI)	*p*-value†	Hazard ratio (95% CI)	*p*-value†	Hazard ratio (95% CI)	*p*-value†
HSV or VZV infection	No	475,884 (63.3%)	1 (ref)	< 0.0001	1 (ref)	< 0.0001	1 (ref)	< 0.0001 0.0001 0.0001
	HSV only VZV only Co-infection	91,990 (12.2%) 113,645 (15.1%) 70,686 (9.4%)	1.38 (1.33–1.43) 1.41 (1.37–1.46) 1.57 (1.50–1.63)		1.49 (1.42–1.56) 1.52 (1.46–1.57) 1.75 (1.66–1.85)		1.46 (1.24–1.72) 1.34 (1.16–1.54) 1.52 (1.24–1.87)	
Age (years)	< 65	537,743 (71.5%)	1 (ref)	< 0.0001	1 (ref)	< 0.0001	1 (ref)	< 0.0001
	≥ 65	214,462 (28.5%)	14.03 (13.64–14.44)		14.70 (14.18–15.24)		8.26 (7.37–9.26)	
Sex	Male Female	348,125 (46.3%) 404,080 (53.7%)	1 (ref) 1.49 (1.46–1.53)	< 0.0001	1 (ref) 1.61 (1.57–1.66)	< 0.0001	1 (ref) 1.21 (1.10–1.34)	0.0002
Depression		58,961 (7.8%)	1.40 (1.36–1.44)	< 0.0001	1.41 (1.36–1.46)	< 0.0001	1.30 (1.12–1.51)	0.0005
Dyslipidaemia		137,800 (18.3%)	0.94 (0.91–0.96)	< 0.0001	0.93 (0.90–0.96)	< 0.0001	0.89 (0.79–1.01)	0.07
Ischaemic stroke		30,950 (4.1%)	1.54 (1.49–1.59)	< 0.0001	1.44 (1.38–1.51)	< 0.0001	2.16 (1.86–2.52)	< 0.0001
Coronary heart disease		86,082 (11.4%)	1.10 (1.07–1.13)	< 0.0001	1.11 (1.07–1.15)	< 0.0001	1.02 (0.89–1.17)	0.77
Hypertension		239,976 (31.9%)	1.39 (1.35–1.42)	< 0.0001	1.37 (1.33–1.41)	< 0.0001	1.76 (1.57–1.97)	< 0.0001
Diabetes mellitus		157,642 (21%)	1.31 (1.28–1.34)	< 0.0001	1.30 (1.26–1.34)	< 0.0001	1.34 (1.20–1.49)	< 0.0001
Chronic kidney disease		3,287 (0.4%)	1.10 (0.96–1.25)	0.16	1.05 (0.89–1.25)	0.55	0.92 (0.48–1.78)	0.80
Body mass index	Underweight	15,993 (2.1%)	1 (ref)	< 0.0001	1 (ref)	< 0.0001	1 (ref)	< 0.0001
	Normal	220,444 (29.3%)	0.66 (0.63–0.69)		0.62 (0.58–0.66)		0.59 (0.47–0.74)	
	Overweight	169,660 (22.6%)	0.54 (0.51–0.56)		0.50 (0.47–0.53)		0.45 (0.36–0.57)	
	Obese	226,268 (30.1%)	0.46 (0.44–0.49)		0.42 (0.40–0.45)		0.45 (0.36–0.57)	
Household income level	Low	138,623 (18.4%)	1 (ref)	< 0.0001	1 (ref)		1 (ref)	
	Lower middle	136,824 (18.2%)	0.96 (0.93–0.99)		0.97 (0.93–1.01)	0.13	0.95 (0.81–1.11)	0.52
	Higher middle	181,648 (24.1%)	0.88 (0.86–0.91)		0.89 (0.85–0.92)	< 0.0001	0.83 (0.72–0.96)	0.01
	High	255,307 (33.9%)	0.88 (0.86–0.91)		0.90 (0.86–0.93)	< 0.0001	0.77 (0.68–0.89)	0.0002

CI, confidence interval; VZV, varicella zoster virus; HSV, herpes simplex virus; ref, reference

†Adjusted for age, sex, VZV & HSV infection, depression, dyslipidaemia, ischaemic stroke, coronary heart disease, hypertension, diabetes mellitus, chronic kidney disease, body mass index, household income level

The comparative results of HSV and VZV infections are described in Supplementary Tables 4 and 5. In the pairwise multivariate analysis, no definite predominance was observed between the HSV-only and VZV-only groups with regards to the risk of dementia. However, the co-infection group had a higher risk than any other group. When the reference was the co-infection group, the risk of dementia was lowest in the no infection group (HR = 0.64, 95% CI:0.61–0.67). The HSV-only group (HR = 0.88, 95% CI:0.83–0.93), and VZV-only group (HR = 0.90, 95% CI:0.86–0.95) had lower risk of dementia than the co-infection group.

### Association of viral infection subtypes and dementia

The number of patients with HSV and VZV infections classified into four categories (eye, CNS, simple, and complicated) are shown in Table 1. The number of patients with VZV infection was higher than those with HSV infection, and CNS involvement in HSV infection was rare (*N* = 64).

Among the four HSV infection categories (Supplementary Table 6), eye (HR = 1.45, 95% CI:1.35–1.56), complicated (HR = 1.48, 95% CI:1.32–1.65), and simple infection (HR = 1.40, 95% CI:1.36–1.45) had a significantly higher risk of dementia than the no infection group. The CNS category had the highest risk (HR = 2.07, 95% CI:0.67–6.42), although the results were not significant. Despite this lack of significance, the CNS category appears to have tendency to increase the risk of dementia when compared to other diagnosis categories (HR = 1.43, 95% CI:0.46–4.44 compared to eye infection, HR = 1.40, 95% CI:0.45–4.37 compared to complicated infection, and HR = 1.47, 95% CI:0.48–4.57 compared to simple infection). The risk for dementia was not predominant in any of the four HSV infection categories.

Among the four VZV infection categories (Supplementary Table 7), eye (HR = 1.59, 95% CI:1.39–1.82), CNS (HR = 1.79, 95% CI:1.71–1.87), complicated (HR = 1.64, 95% CI:1.56–1.73), and simple infection (HR = 1.24, 95% CI:1.20–1.29) showed higher risk of dementia than the non-infection group. However, the risk of simple infection was significantly lower than that of eye, CNS, and complicated VZV infections (*p* < 0.0001).

### Association of viral infection and dementia subtypes

In multivariate analysis based on the type of dementia, a similar trend was observed for all dementia types, including AD and VD (Table 2). The HSV infection was associated with the increased risk of AD (HR = 1.49, 95% CI:1.42–1.56) and VD (HR = 1.46, 95% CI:1.24–1.72) and VZV infection showed increased risk of AD (HR = 1.52, 95% CI:1.46–1.57) and VD (HR = 1.34, 95% CI:1.16–1.54). The co-infection group showed the highest risk of AD (HR = 1.75, 95% CI:1.66–1.85) and VD (HR = 1.52, 95% CI:1.24–1.87).

### Other variables and dementia

In the multivariate analysis, age ≥ 65 years (HR = 14.03, 95% CI:13.64–14.44) and female sex (HR = 1.49, 95% CI:1.46–1.53) were associated with an increased risk of dementia (Table 2). Among systemic comorbidities, depression (HR = 1.40, 95% CI:1.36–1.44), ischaemic stroke (HR = 1.54, 95% CI:1.49–1.59), CHD (HR = 1.10, 95% CI:1.07–1.13), HTN (HR = 1.39, 95% CI:1.35–1.42), and DM (HR = 1.31, 95% CI:1.28–1.34) showed association with the increased risk of dementia. Increasing BMI was associated with a decreasing risk of dementia. The risk of dementia was the highest in the low-household income group.

### Sensitivity analysis

As shown in Figs. 1 and 752,205 participants were included in the study, and 47,476 patients diagnosed with HSV or VZV without corresponding medication within one month were excluded from the analysis. We performed additional analyses in the same manner, including the non-medicated group, for a total of 799,681 patients (Supplementary Table 8). Both HSV (HR = 1.204; CI, 1.14–1.27) and VZV (HR = 1.40; CI, 1.34–1.47) significantly increased the risk of dementia in multivariable analysis after adjusting for age, sex, depression, dyslipidaemia, ischaemic stroke, CHD, HTN, DM, CKD, BMI, and insurance premium level. Furthermore, we conducted sensitivity analyses which corrects for age as a continuous linear or non-linear variables using another cohort (Supplementary Table 9). It also showed that adjusted hazard ratios for HSV and VZV does not dramatically vary across the models considering various non-linear association between age and dementia.

## Discussion

In this nationwide population-based study, the effects of HSV or VZV infection on dementia were comprehensively investigated. The study results showed that HSV and VZV infections were significantly associated with the increased risk of dementia independent of possible confounding factors including age, sex, depression, dyslipidemia, ischemic stroke, CHD, HTN, DM, CKD, BMI, and household income level. The VZV only group showed higher risk of dementia (HR = 1.41) than the HSV only group (H = 1.38), but the difference was not significant. The co-infection group had the highest risk of dementia (H = 1.57) and had a short affected-time (4.09 ± 3.02 years). Compared to single-infection groups, the co-infection group further increased risk of dementia significantly (HR = 1.11, *p*-value < 0.0001 against VZV only group; HR = 1.13, *p*-value < 0.0001 against HSV only group) (Supplementary table 5). VZV infection involving eye or CNS showed higher risk of dementia than simple infection, although simple infection showed higher risk compared with the non-infection group. In HSV, all four types of infection including eye, CNS, complicated, and simple type showed higher risk compared with the non-infection group and the risk of dementia was not predominant in any of the four HSV infection categories.

Whether HSV or VZV infections are associated with an increased risk for dementia remains controversial. In a cohort study conducted in Taiwan with 33,448 participants, the adjusted HR of dementia in the HSV infected group was 2.56 (95% CI:2.35–2.80, *p* < 0.001) [27]. In another national cohort study in Korea including 229,594 individuals, untreated VZV had increased risk of dementia (adjusted HR = 1.12, 95% CI:1.05–1.19). However, the treated group showed an inverse association with dementia (adjusted HR = 0.76, 95% CI:0.65 ~ 0.90) [15]. Similarly, a recent national cohort study conducted in Sweden involving 265,172 patients, reported that patients with a diagnosis of HSV or VZV without antiviral treatment had an adjusted HR of 1.50 (95% CI:1.29–1.74) for dementia. However, the risk decreased in those who received treatment, with an HR of 0.75 (95% CI:0.68–0.83) indicating a negative association with dementia [13]. The result of our study might seem to contradict those of previous studies, but it is due to different definition of variables of each study. In the previous studies, the group of patients with herpes diagnosis and antiviral treatment was compared to patients of herpes diagnosis without antiviral treatment. On the contrary, we defined HSV or VZV infections as those with not only ICD-10 codes but also viral treatment records. Therefore, viral infection groups were compared to non-patient group in this study. In our study, both HSV and VZV infections were associated with an increased risk of dementia. The result of this study is not contradicting the previous ones but highlighting the strong effect of viral infection despite the treatment.

In this study, we defined the exposure variable as viral infections requiring treatment. It is probable that the ICD-10 diagnostic code alone, without any prescribed medication, is primarily used for cases in which there is suspicion but no confirmed diagnosis. In such instances, diagnostic codes are often utilised to facilitate diagnostic tests covered by the NHIS. Therefore, we defined viral infection using both the diagnosis and treatment codes in this study. In South Korea, all types of antiviral agents must be prescribed after seeing a doctor. Therefore, we included any type of medication, not just systemic (intravenous or oral) medications but also topical drugs such as eye drops or ointments.

The total number of HSV and VZV infections treated with medication was 162,676 (21.6%) and 184,331 (24.5%), respectively. When comparing the findings to those of a Swedish cohort [13], the difference in the number of patients visiting hospitals and receiving antiviral treatment between VZV and HSV was not significant because of South Korea’s accessible and affordable medical services. The Swedish study reported 24,045 patients who received antiviral treatment for VZV and 6,510 patients for HSV [13]. Although it is apparent that HSV is more prevalent than VZV, based on clinical observations, the reported number of VZV cases is higher than that of HSV cases. As zoster infection is usually accompanied by pain, most patients with VZV infection visit the hospital. However, in numerous cases, individuals with mild blistering caused by the HSV do not seek medical attention because of the absence of severe pain. Therefore, we cannot be certain whether the recorded number of patients with HSV accurately reflects the actual number of patients. This inherent limitation causes selection bias.

Our study revealed that HSV and VZV co-infection was associated with a higher risk of dementia than HSV or VZV alone. Furthermore, dementia in the co-infection group showed the longest follow-up and the shortest period from viral infection to dementia. Viral co-infection led to the development of dementia in a short period of time. This may be because of the stronger effect of viral co-infection on the development of dementia, or because there was little time between the second infection and the end of follow-up in the co-infection group. Trigeminal ganglion biopsies of cadavers revealed that HSV and VZV could reside in the same trigeminal nerve [28]. In an experimental study, human embryonic stem cell-derived neurons were infected with HSV and VZV either simultaneously or consecutively [29]. Therefore, the interaction between two viruses residing in the same neuron could increase the risk of dementia development or simply be due to the additive effects of the viruses.

Among the four subtypes of HSV infection (eye, CNS, complicated, and simple), only CNS involvement demonstrated an insignificant risk for dementia (HR = 2.07, 95% CI:0.67–6.42). However, it is noteworthy that the estimated HR value for the CNS group was the highest, indicating a potential tendency for an increased risk of dementia compared to the other diagnosis categories, although this difference was not significant. These findings are clinically important and warrant further investigation.

Simple VZV infection was associated with a lower risk of dementia than other types of VZV infection, although the risk was still higher than that in the non-infection group. We found that the risk of dementia was not significantly different between the eye and CNS VZV infection group (*p* = 0.10).

In this study, simple viral infections (both HSV and VZV) were associated with an increased risk of dementia. Recently, an observational study conducted in Korea discussed that HSV1 infections, such as the oral (adjusted HR = 1.27, 95% CI:1.23 ~ 1.32) or ocular (adjusted HR = 1.20, 95% CI:1.15 ~ 1.25) subtypes, were associated with dementia [30]. Recent research has suggested that peripheral nerve function impairment may be associated with cognitive impairment [31, 32]. Furthermore, decreased retinal nerve fibre layer thickness in cognitive impairment has been confirmed in a meta-analysis, showing an association between peripheral nerves and dementia [33]. Therefore, the peripheral nerve damage caused by viral infections may be associated with dementia [33]. However, further studies are required to determine the mechanisms underlying the results of our study.

The large cohort size, based on a national database, is a strength of this study. A comprehensive analysis was performed to assess the risk of HSV and VZV infections, and their combined risk. We considered HSV and VZV as time-varying variables to reflect changes in status during the follow-up period in the multivariable analyses. We strictly defined the exposure variable as a treatment-requiring viral infection based on diagnosis codes and drug prescription history within one month. Furthermore, HSV and VZV infections were subdivided into eye, CNS, complicated, and simple subgroups for risk comparison. The effects were analysed according to the type of dementia.

Since infection of HSV or VZV is associated with increased risk of dementia, prevention of these infections would be important in future public health. It is known that transmission of HSV occurs through secretions from symtomatic or asymptomatic individuals (cutaneous or sexual) [34, 35]. Therefore, hand hygiene and condom use in sexual intercourse should be recommended. As VZV is highly contagious and mostly transmitted via cutaneous vesicles, healthcare workers should beware of increased risk [36, 37]. Moreover, vaccination would prevent the infection and reduce the associated risk of dementia [38]. The result of the study also implies that dementia development would be affected by pathophysiologic mechanism of immune system against viral infection [39].

### Limitations

Our study had several limitations. First, South Korea is a single-race nation, and the Korean population comprises mostly a homogenous Northeast Asian ethnicity. Further studies including other ethnicities are required to validate our results. Second, we did not focus on a more detailed analysis of types of dementia other than AD and VD. Third, the observational study design did not allow for any causal inference; therefore, we only demonstrated associations. Fourth, potential risk factors for dementia, such as genetic factors or vaccinations [40–42] could not be analysed in this study because such information was not available in the NHIS data. Nevertheless, the authors attempted to adjust for as many confounding factors as possible. Fifth, adjusting age as a dichotomous variable may cause considerable confounding. Nevertheless, the authors performed sensitivity analyses using another NHIS cohort, which shows the robustness of HSV and VZV infection effect on the risk of dementia across the models considering various non-linear association between age and dementia. Finally, to minimise the inclusion of false-positive subjects, we rigorously defined the exposure variable as a treatment-requiring viral infection. We acknowledge that this approach may introduce potential misclassification or selection bias. Moreover, our strict definition could potentially result in an overestimation of the effects, as this definition might exclude mild cases. Nevertheless, previous research has indicated that antiviral treatments mitigate the impact of viral infections on dementia [13–15]. Nonetheless, our study reveals a noteworthy correlation between viral infections that necessitate treatment and the occurrence of dementia. Furthermore, these findings remained reliable in our sensitivity analysis, which included encompassed instances where viral infections were diagnosed but medication was not administered as the exposure variable.

## Conclusion

In conclusion, HSV and VZV infections are associated with an increased risk of dementia. Patients experiencing co-infection with HSV and VZV, complicated VZV infection, or VZV involved in eye or CNS infections have a higher risk of dementia. Physicians should be aware of these associations in patients with viral infections. Further research on the mechanisms underlying viral infections and dementia is required.

### Electronic supplementary material

Below is the link to the electronic supplementary material.

Supplementary Material 1

## Data Availability

The data that support the findings of this study are from medical records of Korean National Health Insurance Service and were used under license for the current study, so are not publicly available.

## Abbreviations

AD: Alzheimer’s disease
HSV: Herpes simplex virus
VZV: Varicella zoster virus
HR: Hazard ratio
CNS: Central nervous system
NHIS: National Health Insurance Service
ICD-10: International Classification of Disease 10th revision
BMI: Body mass index
VD: Vascular dementia
HTN: Hypertension
DM: Diabetes mellitus
CKD: Chronic kidney disease
CHD: Coronary heart disease
SD: Standard deviation
CI: Confidence interval
PCR: Polymerase chain reaction
CSF: Cerebrospinal fluid
HZO: Herpes zoster ophthalmicus
