# Supplementary material for: The associations of herpes simplex virus and varicella zoster virus infection with dementia: a nationwide retrospective cohort study
Source: Alzheimers Res Ther. 2024 Mar 12;16:57. doi: 10.1186/s13195-024-01418-7 (PMC10935826; doi:10.1186/s13195-024-01418-7)
Supplement: Supplementary file 1 — Supplementary Material 1 [file 13195_2024_1418_MOESM1_ESM.pdf]

Supplement table 1. ICD-10 codes used to define exposure, outcome, and co-variables*

**HSV**		
	B00.5	Herpesviral ocular disease
	H03.1	Involvement of eyelid in other infectious diseases classified elsewhere
	H13.1	Conjunctivitis in infectious and parasitic diseases classified elsewhere
	H19.1	Herpesviral keratitis and keratoconjunctivitis
	H22.0	Iridocyclitis in infectious and parasitic diseases classified elsewhere
	B00.3	Herpesviral meningitis
	B00.7	Disseminated herpesviral disease
	B00.8	Other forms of herpesviral infection
	B00	Herpesviral [herpes simplex] infections
	B00.0	Eczema herpeticum
	B00.1	Herpesviral vesicular dermatitis
	B00.9	Herpesviral infection, unspecified
	A60*	Anogenital herpesviral [herpes simplex] infection
	B002	Herpesviral gingivostomatitis and pharyngotonsillitis
**VZV**		
	B02.3	Zoster ocular disease
	H03.1	Involvement of eyelid in other infectious diseases classified elsewhere
	H13.1	Conjunctivitis in infectious and parasitic diseases classified elsewhere
	H19.0	Scleritis and episcleritis in diseases classified elsewhere
	H19.2	Keratitis and keratoconjunctivitis in other infectious and parasitic diseases classified elsewhere
	H22.0	Iridocyclitis in infectious and parasitic diseases classified elsewhere
	B02.1	Zoster meningitis
	B02.2	Zoster with other nervous system involvement
	B02.7	Disseminated zoster
	B02.8	Zoster with other complications
	B02	Zoster [herpes zoster]
	B02.9	Zoster without complications
**Dementia**		
	F00	Dementia in Alzheimer’s disease
	F01	Vascular dementia
	F02	Dementia in other diseases classified elsewhere
	F03	Unspecified dementia
	G30	Alzheimer’s disease

G31.00	Frontotemporal dementia
**Others**	
I10	Essential (primary) hypertension
I11	Hypertensive heart disease
I12	Hypertensive renal disease
I13	Hypertensive heart and renal diseases
I15	Secondary hypertension
E10	Type 1 diabetes mellitus
E11	Type 2 diabetes mellitus
E12	Malnutrition-related diabetes mellitus
E13	Other specified diabetes mellitus
E14	Unspecified diabetes mellitus
E78.4	Other hyperlipidaemia
E78.5	Hyperlipidaemia, unspecified
E78.0	Pure hypercholesterolaemia
I70	Atherosclerosis
I63	Cerebral infarction
I64	Stroke, not specified as haemorrhage or infarction
I65	Occlusion and stenosis of precerebral arteries, not resulting in cerebral infarction
I66	Occlusion and stenosis of cerebral arteries, not resulting in cerebral infarction
I20	Angina pectoris
I21	Acute myocardial infarction
I22	Subsequent myocardial infarction
I23	Certain current complication following acute myocardial infarction
I24	Other acute ischaemic heart disease
I25	Chronic ischaemic heart disease
F32	Depressive episode
F33	Recurrent depressive disorder
F34	Persistent mood [affective] disorders
N18	Chronic kidney disease

HSV, herpes simplex virus; VZV, varicella zoster virus.

*Since the purpose of our study was not to investigate acute changes in viral infection, ICD-10 codes for B00.4 (herpesviral meningoencephalitis) and B02.0 (zoster meningoencephalitis), and F05 (delirium, not induced by alcohol and other psychoactive substances) were excluded to define the exposure variable (viral infection) and outcome variable (dementia). Direct viral invasion of the brain can cause cognitive dysfunction, including delirium, and it is a reversible change that can be recovered when the viral infection is treated.

Supplement table 2. Working definition of each variable

Viral infection [¥]	Category	ICD-10 diagnostic code
**HSV**	**Eye**	B00.5 only or with H03.1, H13.1, H19.1, H22.0
	**CNS**	B00.3
	**Complicated**	B00.7, B00.8
	**Simple***	B00, B00.0, B00.1, B00.9, A60, B00.2
**VZV**	**Eye**	B02.3 only or with H03.1, H13.1, H19.0 H19.2, H22.0
	**CNS**	B02.1, B02.2
	**Complicated**	B02.7, B02.8
	**Simple***	B02, B02.9
**Dementia subtype [£]**		
**AD**		F00, G30 only or with F03
**VD**		F01 only or with F03
**Other co-variables**		
**HTN**		I10, I11, I12, I13, I14, I15
**DM**		E10, E11, E12, E13, E14
**Dyslipidaemia**		E78.4, E78.5, E78.0, I70
**Ischaemic stroke**		I63, I64, I65, I66
**CHD**		I20, I21, I22, I23, I24, I25
**CKD[§]**		N18

**Depression**	F32, F33, F34

HSV, herpes simplex virus; VZV, varicella zoster virus; CNS, central nervous system; AD, Alzheimer dementia; VD, vascular dementia; HTN, hypertension; DM, diabetes mellitus; CHD, coronary heart disease; CKD, chronic kidney disease.

[¥] Each viral infection was defined as ICD-10 diagnostic codes with antiviral medicine prescription within a month of diagnosis.

*Simple skin or genital viral infection was included. Only those diagnostic codes without any other codes combined.

[£] Each subtype of dementia was defined by the first prescription of an acetylcholinesterase inhibitor or NMDA receptor antagonist with an ICD-10 code for Alzheimer's disease (F00, G30) and vascular dementia (F01). An additional ICD-10 code for unspecified dementia (F03) is permitted for each category of AD or VD.

[§]CKD was defined as any of the following three criteria: (1) glomerular filtration rate < 60 mL/min/1.73 m^2; (2) ICD-10 code N18; and (3) rare intractable disease codes for CKD (V001, V003) or regular dialysis codes (O7202, O7062, O7093).

Supplement table 3. Impact of viral infection on the risk of dementia via univariate analysis of variables

Variables		Cohort (N = 752,205)	Hazard ratio (95% CI)	p-value
**Age (years)**	< 65	537,743 (71.5%)	1 (ref)	< 0.0001
	≥ 65	214,462 (28.5%)	19.72 (19.25–20.21)	
**Sex**	Male	348,125 (46.3%)	1 (ref)	< 0.0001
	Female	404,080 (53.7%)	2.14 (2.10–2.18)	
**HSV infection**		162,676 (21.6%)	1.20 (1.17–1.22)	< 0.0001
**VZV infection**		184,331 (24.5%)	1.57 (1.54–1.60)	< 0.0001
**HSV or VZV infection**	No	475,884 (63.3%)	1 (ref)	< 0.0001
	HSV only	91,990 (12.2%)	1.16 (1.13–1.20)	
	VZV only	113,645 (15.1%)	1.62 (1.58–1.66)	
	Co-infection	70,686 (9.4%)	1.54 (1.49–1.60)	
**Depression**		58,961 (7.8%)	2.14 (2.09–2.19)	< 0.0001
**Dyslipidaemia**		137,800 (18.3%)	1.60 (1.57–1.63)	< 0.0001
**Ischaemic stroke**		30,950 (4.1%)	3.72 (3.62–3.83)	< 0.0001
**Coronary heart disease**		86,082 (11.4%)	2.19 (2.15–2.24)	< 0.0001
**Hypertension**		239,976 (31.9%)	3.06 (3.01–3.11)	< 0.0001
**Diabetes mellitus**		157,642 (21%)	2.26 (2.21–2.30)	< 0.0001
**Chronic kidney disease**		3,287 (0.4%)	2.12 (1.92–2.35)	< 0.0001
**Body mass index**	Underweight	15,993 (2.1%)	1 (ref)	< 0.0001
	Normal	220,444 (29.3%)	0.45 (0.43–0.47)	
	Overweight	169,660 (22.6%)	0.37 (0.35–0.38)	
	Obese	226,268 (30.1%)	0.35 (0.33–0.37)	
**Household income level**	Low	138,623 (18.4%)	1 (ref)	< 0.0001
	Lower middle	136,824 (18.2%)	0.86 (0.83–0.89)	
	Higher middle	181,648 (24.1%)	0.90 (0.87–0.92)	
	High	255,307 (33.9%)	1.03 (1.01–1.06)	

CI, confidence interval; ref, reference; VZV, varicella zoster virus; HSV, herpes simplex virus.

*Univariate Cox proportional hazards regression analysis was used to calculate crude hazard ratios (HRs) and 95% confidence intervals (CIs) for dementia.

Supplement table 4. Univariate analysis of pairwise hazard ratio of viral infection on dementia

Variables		Hazard ratio 1 (95% CI)	p-value	Hazard ratio 2 (95% CI)	p-value	Hazard ratio 3 (95% CI)	p-value	Hazard ratio 4 (95% CI)	p-value
**HSV or VZV infection**	No	1 (ref)	.	0.86 (0.83–0.89)	**< 0.0001**	0.62 (0.60–0.63)	**< 0.0001**	0.65 (0.63–0.67)	**< 0.0001**
	HSV only	1.16 (1.13–1.20)	**< 0.0001**	1 (ref)	.	0.72 (0.69–0.74)	**< 0.0001**	0.75 (0.72–0.79)	**< 0.0001**
	VZV only	1.62 (1.58–1.66)	**< 0.0001**	1.40 (1.35–1.45)	**< 0.0001**	1 (ref)	.	1.05 (1.01–1.10)	**0.02**
	Co-infection	1.54 (1.49–1.60)	**< 0.0001**	1.33 (1.27–1.39)	**< 0.0001**	0.95 (0.91–0.99)	**0.02**	1 (ref)	.

CI, confidence interval; VZV, varicella zoster virus; HSV, herpes simplex virus; ref, reference.

*Univariate Cox proportional hazards regression analysis was used to calculate crude hazard ratios (HRs) and 95% confidence intervals (CIs) for dementia.

Supplement table 5. Multivariate analysis of pairwise hazard ratio of viral infection on dementia

Variables		Hazard ratio 1 † (95% CI)	p-value	Hazard ratio 2† (95% CI)	p-value	Hazard ratio 3† (95% CI)	p-value	Hazard ratio 4† (95% CI)	p-value
**HSV or VZV infection**	No	1 (ref)	.	0.72 (0.70–0.75)	**< 0.0001**	0.71 (0.69–0.73)	**< 0.0001**	0.64 (0.61–0.67)	**< 0.0001**
	HSV only	1.38 (1.33–1.43)	**< 0.0001**	1 (ref)	.	0.98 (0.94–1.02)	0.33	0.88 (0.84–0.93)	**< 0.0001**
	VZV only	1.41 (1.37–1.46)	**< 0.0001**	1.02 (0.98–1.07)	0.3289	1 (ref)	.	0.90 (0.86–0.95)	**< 0.0001**
	Co-infection	1.57 (1.50–1.63)	**< 0.0001**	1.13 (1.07–1.20)	**< 0.0001**	1.11 (1.06–1.16)	**< 0.0001**	1 (ref)	.

CI, confidence interval; VZV, varicella zoster virus; HSV, herpes simplex virus; ref, reference.

†Adjusted for age, sex, depression, dyslipidaemia, ischaemic stroke, coronary heart disease, hypertension, diabetes mellitus, chronic kidney disease, body mass index, insurance premium level.

Supplement table 6. Multivariate analysis of pairwise hazard ratio of herpes simplex virus infection on dementia

		Hazard ratio 1† (95% CI)	p-value	Hazard ratio 2† (95% CI)	p-value	Hazard ratio 3† (95% CI)	p-value	Hazard ratio 4† (95% CI)	p-value	Hazard ratio 5† (95% CI)	p-value
**HSV infection**	None	1 (ref)	.	0.69 (0.64–0.74)	**< 0.0001**	0.48 (0.16–1.50)	0.21	0.68 (0.60–0.76)	**< 0.0001**	0.71 (0.69–0.74)	**< 0.0001**
	eye	1.45 (1.35–1.56)	**< 0.0001**	1 (ref)	.	0.70 (0.23–2.17)	0.54	0.98 (0.86–1.12)	0.77	1.03 (0.95–1.11)	0.43
	CNS	2.07 (0.67–6.42)	0.21	1.43 (0.46–4.44)	0.54	1 (ref)	.	1.40 (0.45–4.37)	0.56	1.47 (0.48–4.57)	0.50
	comp	1.48 (1.32–1.65)	**< 0.0001**	1.02 (0.89–1.17)	0.76	0.71 (0.23–2.23)	0.56	1 (ref)	.	1.05 (0.94–1.18)	0.39
	simple	1.40 (1.36–1.45)	**< 0.0001**	0.97 (0.90–1.05)	0.43	0.68 (0.22–2.10)	0.50	0.95 (0.85–1.07)	0.39	1 (ref)	.

CI, confidence interval; HSV, herpes simplex virus; ref, reference; CNS, central nervous system; comp, complicated

†Adjusted for age, sex, depression, dyslipidaemia, ischaemic stroke, coronary heart disease, hypertension, diabetes mellitus, chronic kidney disease, body mass index, insurance premium level.

Supplement table 7. Multivariate analysis of pairwise hazard ratio of varicella zoster virus infection on dementia

		Hazard ratio 1† (95% CI)	p-value	Hazard ratio 2† (95% CI)	p-value	Hazard ratio 3† (95% CI)	p-value	Hazard ratio 4† (95% CI)	p-value	Hazard ratio 5† (95% CI)	p-value
**VZV infection**	None	1 (ref)	.	0.63 (0.55–0.72)	**< 0.0001**	0.56 (0.54–0.58)	**< 0.0001**	0.61 (0.58–0.64)	**< 0.0001**	0.81 (0.78–0.83)	**< 0.0001**
	eye	1.59 (1.39–1.82)	**< 0.0001**	1 (ref)	.	0.89 (0.77–1.02)	0.10	0.97 (0.84–1.12)	0.68	1.28 (1.12–1.47)	**0.0004**
	CNS	1.79 (1.71–1.87)	**< 0.0001**	1.12 (0.98–1.29)	0.10	1 (ref)	.	1.09 (1.02–1.16)	**0.01**	1.44 (1.36–1.52)	**< 0.0001**
	comp	1.64 (1.56–1.73)	**< 0.0001**	1.03 (0.89–1.19)	0.68	0.92 (0.86–0.98)	**0.01**	1 (ref)	.	1.32 (1.24–1.40)	**< 0.0001**
	simple	1.24 (1.20–1.29)	**< 0.0001**	0.78 (0.68–0.90)	**0.0004**	0.70 (0.66–0.73)	**< 0.0001**	0.76 (0.71–0.80)	**< 0.0001**	1 (ref)	.

CI, confidence interval; VZV, varicella zoster virus; ref, reference; CNS, central nervous system; comp, complicated.

†Adjusted for age, sex, depression, dyslipidaemia, ischaemic stroke, coronary heart disease, hypertension, diabetes mellitus, chronic kidney disease, body mass index, insurance premium level.

Supplement table 8. Sensitivity analysis including viral infection for the diagnosis group without medication

		All dementia	
**Variables**		**Hazard ratio (95% CI)**	**p-value†**
**Age (years)**	< 65	1 (ref)	< 0.0001
	≥ 65	14.68 (14.28–15.08)	
**Sex**	Male	1 (ref)	< 0.0001
	Female	1.46 (1.43–1.50)	
**HSV infection**	No	1 (ref)	< 0.0001
	HSV infection	1.20 (1.14–1.27)	
**VZV infection**	No	1 (ref)	< 0.0001
	VZV infection	1.40 (1.34–1.47)	
**Depression**		1.41 (1.37–1.45)	< 0.0001
**Dyslipidaemia**		0.93 (0.91–0.96)	< 0.0001
**Ischaemic stroke**		1.55 (1.50–1.60)	< 0.0001
**Coronary heart disease**		1.10 (1.07–1.13)	< 0.0001
**Hypertension**		1.40 (1.37–1.43)	< 0.0001
**Diabetes mellitus**		1.33 (1.30–1.36)	< 0.0001
**Chronic kidney disease**		1.12 (1.00–1.27)	
**Body mass index**	Underweight	1 (ref)	< 0.0001
	Normal	0.64 (0.61–0.67)	
	Overweight	0.52 (0.49–0.54)	
	Obese	0.45 (0.42–0.47)	
**Household income level**	Low	1 (ref)	< 0.0001
	Lower middle	0.96 (0.94–0.99)	
	Higher middle	0.88 (0.86–0.91)	
	High	0.89 (0.86–0.91)	

CI, confidence interval; VZV, varicella zoster virus; HSV, herpes simplex virus; ref, reference.

Supplement table 9. Sensitivity analysis of various non-linear association between age and dementia in another NHIS cohort

		Model 1[a]		Model 2[b]		Model 3[c]	
**Variables**		**Hazard ratio (95% CI)**	**p-value[d]**	**Hazard ratio (95% CI)**	**p-value[d]**	**Hazard ratio (95% CI)**	**p-value[d]**
**HSV or VZV infection**	No	1 (ref)	< 0.0001	1 (ref)	< 0.0001	1 (ref)	< 0.0001
	HSV only	1.17 (1.16-1.18)		1.16 (1.12-1.19)		1.16 (1.12-1.19)	
	VZV only	1.07 (1.06-1.07)		1.06 (1.04-1.09)		1.06 (1.04-1.09)	
	Co-infection	1.24 (1.22-1.25)		1.26 (1.21-1.31)		1.26 (1.21-1.31)	
**Age, years[e]**		1.11 (1.11-1.11)	< 0.0001	1.24 (1.23-1.26)	< 0.0001	1.11 (1.11-1.11)[e]	< 0.0001
**Sex**	Male	1 (ref)	< 0.0001	1 (ref)	< 0.0001	1 (ref)	< 0.0001
	Female	1.43 (1.42-1.43)		1.46 (1.43-1.48)		1.46 (1.43-1.48)	
**Depression**		1.56 (1.55-1.57)	< 0.0001	1.55 (1.51-1.59)	< 0.0001	1.55 (1.51-1.59)	< 0.0001
**Dyslipidaemia**		1.03 (1.02-1.03)	< 0.0001	1.02 (1-1.04)	0.093	1.02 (1-1.04)	0.089
**Ischaemic stroke**		1.48 (1.46-1.49)	< 0.0001	1.46 (1.42-1.5)	< 0.0001	1.46 (1.42-1.5)	< 0.0001
**Coronary heart disease**		1.11 (1.1-1.11)	< 0.0001	1.11 (1.08-1.13)	< 0.0001	1.11 (1.08-1.13)	< 0.0001
**Hypertension**		1.06 (1.06-1.07)	< 0.0001	1.05 (1.03-1.07)	< 0.0001	1.05 (1.03-1.07)	< 0.0001
**Diabetes mellitus**		1.21 (1.21-1.22)	< 0.0001	1.21 (1.19-1.24)	< 0.0001	1.21 (1.19-1.24)	< 0.0001
**Chronic kidney disease**		1.19 (1.15-1.23)	< 0.0001	1.19 (1.06-1.34)	0.004	1.19 (1.06-1.34)	0.0038
**Body mass index**	Underweight	1 (ref)	< 0.0001	1 (ref)	< 0.0001	1 (ref)	< 0.0001
	Normal	0.87 (0.86-0.88)		0.87 (0.83-0.91)		0.87 (0.84-0.91)	
	Overweight	0.82 (0.81-0.83)		0.81 (0.78-0.85)		0.81 (0.78-0.85)	
	Obese	0.8 (0.79-0.81)		0.8 (0.76-0.83)		0.8 (0.76-0.83)	
**Household income level**	Low	1 (ref)	< 0.0001	1 (ref)	0.2053	1 (ref)	0.205
	Lower middle	1.01 (1-1.02)	0.011	1.02 (0.99-1.04)	0.227	1.02 (0.99-1.04)	0.23
	Higher middle	1 (0.99-1.01)	0.631	1.02 (0.99-1.04)	0.201	1.02 (0.99-1.04)	0.2
	High	0.95 (0.94-0.95)	< 0.0001	0.98 (0.96-1)	0.104	0.98 (0.96-1)	0.11

CI, confidence interval; VZV, varicella zoster virus; HSV, herpes simplex virus; ref, reference.

The cohort was collected from NHIS database with identical inclusion/exclusion criteria and definition of all variables. HSV and VZV were considered time-varying

variables.

[a] From multivariable cox proportional hazards regression model with age as linear variable.

[b] Further adjusted for age-squared term.

[c] From multivariable cox proportional hazards regression model with age using a penalised spline function.

[d] Adjusted for age, sex, VZV & HSV infection, depression, dyslipidaemia, ischaemic stroke, coronary heart disease, hypertension, diabetes mellitus, chronic kidney disease, body mass index, household income level.

[e] This is the adjusted hazard ratio of age (continuous variable) which assumes the linear association with the risk of dementia. For Model 2, the adjusted hazard ratio of age-squared term (HR = 0.99, 95% CI:0.99-0.99, P-value <0.0001) was not shown. For Model 3, The model showed a significant non-linear association of age on the risk of dementia after adjusting the other variables. And non-linear trend of adjusted hazard ratio which varies across the age values was not shown.
